# Good advice for endothelial cells: Get in line, relax tension, and go with the flow

**DOI:** 10.1063/1.5129812

**Published:** 2020-02-26

**Authors:** Roland Kaunas

**Affiliations:** Department of Biomedical Engineering and Department of Cellular and Molecular Medicine, Texas A&M University, College Station, Texas 77843-3120, USA

## Abstract

Endothelial cells (ECs) are continuously subjected to fluid wall shear stress (WSS) and cyclic strain caused by pulsatile blood flow and pressure. It is well established that these hemodynamic forces each play important roles in vascular disease, but their combined effects are not well understood. ECs remodel in response to both WSS and cyclic strain to align along the vessel axis, but in areas prone to atherogenesis, such an alignment is absent. In this perspective, experimental and clinical findings will be reviewed, which have revealed the characteristics of WSS and cyclic strain, which are associated with atherosclerosis, spanning studies on whole blood vessels to individual cells to mechanosensing molecules. Examples are described regarding the use of computational modeling to elucidate the mechanisms by which EC alignment contributes to mechanical homeostasis. Finally, the need to move toward an integrated understanding of how hemodynamic forces influence EC mechanotransduction is presented, which holds the potential to move our currently fragmented understanding to a true appreciation of the role of mechanical stimuli in atherosclerosis.

## INTRODUCTION

**FIG. 1.**
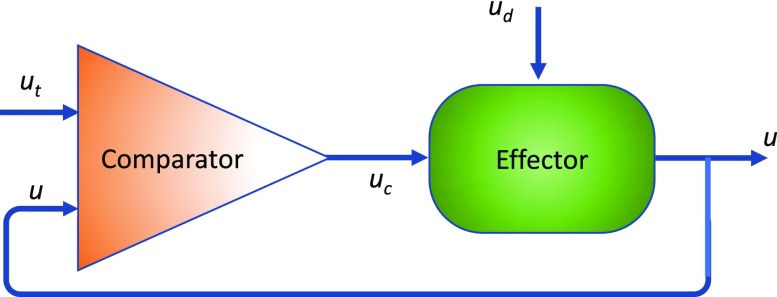
Simple negative feedback loop. A disturbance *u*_d_ results in a deviation of a parameter *u* from the setpoint level *u*_t_. A Comparator senses this deviation to result in a control signal *u*_c_ that is transduced into a compensatory action by the Effector to minimize the deviation.

It is well-known that arterial endothelial cells (ECs) respond to changes in the flow rate to regulate vascular tone in order to maintain constant wall shear stress (WSS). This response can be described in terms of a simple negative feedback loop (Fig. 1) consisting of a comparator that monitors a controlled condition (level of an input *u* relative to a setpoint *u*t) and an effector that receives directions from the comparator and produces a response that changes the controlled condition.^2^ In the case of increased blood flow, the endothelium (comparator) detects an increase in WSS (*u*) above the setpoint level (*u*t) and responds by increasing the rate of nitric oxide (NO) release (*u*c). NO diffuses to the smooth muscle cells (effector) to induce relaxation, thus allowing the blood vessel to dilate and thus reduce the WSS back toward the setpoint level. In the 2003 Walter B. Cannon Award Lecture,^1^ Shu Chien referred to this process as the “Wisdom of the Cell,” as a part of the theories of physiological homeostasis described in Cannon's book “Wisdom of the Body.”

Chien also described in his lecture how the unique fluid mechanical environment in regions of curvature and branch points of the arterial tree contribute to the focal development of atherosclerotic plaques. Here, a failure to achieve mechanical homeostasis compromises the ability of the endothelium to maintain an intact barrier and also activates the ECs to recruit monocytes through expression of pro-inflammatory surface receptors. This lecture explained the roles of not only fluid shear forces but also cyclic stretching of the endothelium in inducing pro-atherosclerotic signal transduction when these mechanical forces lacked directionality. This foundational concept has inspired further developments within the field of mechanobiology, which not only confirm the validity of these ideas but also reveal the complexity of the pro-atherogenic environment. In this perspective, experimental observations that provide further clarity regarding the multiple mechanical factors contributing to arterial endothelial mechanical homeostasis (or lack thereof) will be summarized. Given the complexity of the spatially varying and temporally dynamic environment in arterial flow, the use of mathematical modeling will be emphasized to provide a framework for integrating these factors into a comprehensive understanding.

## PRO-ATHEROGENIC CHARACTERISTICS OF FLUID SHEAR STRESS

The flow in the relatively straight regions of the arterial tree is pulsatile with a marked forward flow, whereas the flow at branch points and curvatures is frequently referred to as “disturbed flow.” Disturbed flow describes a number of complexities in the flow, which include flow reversal, flow separation, regions of very low and regions of very high WSS, high spatial gradients in WSS, and substantial secondary flows. Though there has been substantial debate over the years, the prevailing theory is that low^3,4^ and oscillatory^5^ WSS play key roles in the preferential development of atherosclerosis at arterial bifurcations. This is consistent with the results from studies in animals in which a stenosis is generated using a clip, resulting in substantially diminished EC cell junction integrity and reduced cell elongation in the post-stenotic dilatation site, where the WSS is complex and reciprocating with little net flow.^6^

**FIG. 2.**
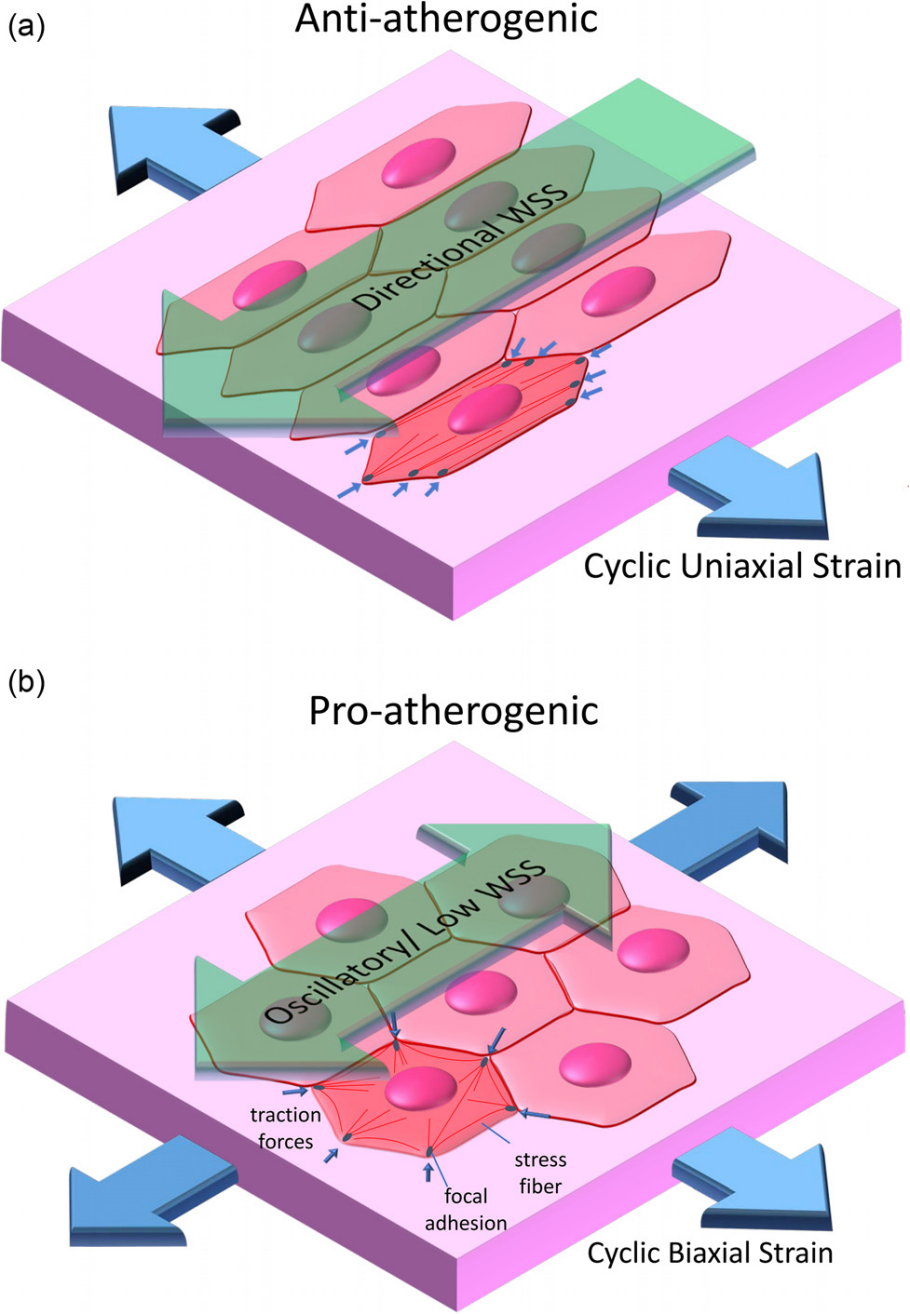
Directional mechanical forces are anti-atherogenic, while non-directional forces are pro-atherogenic. (a) Directional forces (steady or pulsatile WSS with a significant forward component and circumferential cyclic strain) promote the alignment of the EC shape, actin stress fibers and traction forces aligned with the principal axis of the artery. (b) Non-directional forces (oscillatory/low WSS and cyclic equibiaxial strain) do not result in an alignment response. Non-directional forces induce an atherogenic EC phenotype characterized by inflammatory signaling, high cell turnover, and disrupted cell junctions, while directional forces have the opposite effect.

Cell culture systems that apply well-defined WSS waveforms to EC monolayers have provided substantial insight. A parallel-plate flow chamber containing a step to mimic a stenotic region generates flow separation with regions of flow reattachment.^7,8^ This system has been used to show that regions of low and spatially varying WSS result in high EC turnover, disrupted cell junction integrity, and elevated pro-inflammatory adhesion molecule expression.^1^ Applying a time-varying WSS with either a parallel-plate or cone-plate system without a spatial WSS gradient further revealed that oscillatory WSS with large amplitude^6^ or oscillatory WSS with a low amplitude^9^ also results in similar pro-atherogenic EC phenotypes, while a pulsatile, non-reversing WSS has the opposite effect (Fig. 2).

Low/oscillatory WSS only partially describes the complex WSS characteristics found *in vivo*. More recently, additional metrics of WSS have been proposed to capture the complex, multi-directional nature of WSS. Evidence has been reported that such off-axis flow may help to explain the distributions of lesions observed *in vivo*, which are inconsistent with the low/oscillatory WSS paradigm.^10^ Analysis of WSS metrics and lesion distributions at the intercostal branch ostia of rabbit aortae correlated strongly with average magnitude of WSS components acting transversely to the mean vector (transWSS), while the oscillatory shear index (OSI) and time-average WSS did not correlate with lesion prevelance.^11^ The axial component of WSS aligned with the vessel centerline (WSS_ax_) has also been introduced to quantify flow reversal for multiaxial WSS.^12^ Computational modeling of flows in human subject-specific models of carotid bifurcations indicates that transWSS is low and that regions of high OSI and low time-average WSS (TAWSS) are co-localized to regions of flow reversal^13^ where lesions are typically localized. Together, these results suggest that two WSS-based descriptors of the multi-directionality of the flow, transWSS and WSS_ax_, do not replace, but rather complement, OSI and TAWSS in the analysis of the effects of WSS on the EC function in health and disease.^13^

Experimental models provide insight into the ability of ECs to respond to the off-axis WSS. ECs prealigned with micropatterned adhesive strips respond to pulsatile WSS oriented parallel to the strips by enhanced stress fiber formation and reduced apoptosis, which was not observed when the cells were prealigned perpendicular to the direction of WSS.^14^ Using the same approach, Wang *et al.*^15^ demonstrated that oscillatory WSS activates proinflammatory nuclear factor-κB (NF-κB) signaling more so when ECs are prealigned perpendicular, rather than parallel, to the flow direction. Also, they showed that oscillatory WSS phosphorylates anti-atherogenic endothelial nitric oxide synthase (eNOS) in ECs prealigned parallel, but not perpendicular, to the flow direction. To further explore the effect of cell orientation, Wang *et al.*^15^ subjected ECs that had been subjected to 24 h of steady WSS to induce alignment and then subjected to a change in the WSS direction using a flow chamber with a rotatable base capable of changing the flow direction to any angle. Phosphorylation of eNOS was maximal after a 180° change in the direction and undetectable after a 90° change. In contrast, activation of NF-κB was maximal at 90° and undetectable at 180°.

## PRO-ATHEROGENIC CHARACTERISTICS OF CYCLIC STRAIN

Hypertension has long been recognized as a risk factor for atherosclerosis.^16,17^ In particular, the pulsatile component of blood pressure (pulse pressure) has been identified as an independent risk factor for atherosclerosis.^18,19^ Pulse pressure generates cyclic strain that is primarily circumferentially directed in straight arteries and contributes to axial alignment of ECs. The reduction in cyclic strain with an external wrap inhibits hypertension-induced plaque formation^20,21^ and EC alignment^22^ in cholesterol-fed rabbits. The relationship between the distributions of wall strain metrics and regions prone to atherosclerosis is not as well studied as that for WSS. Using a computational model, Thubrikar and Robicsek^23^ predicted tensile stress is high in regions prone to atherogenesis in the human carotid bifurcation. They also measured elevated strains of 5%–7% proximal to the ostium bovine circumflex coronary arterial branch (vs 2%–3% strain further away). Higher resolution strain maps were reported, based on computational analysis of subject-specific carotid bifurcation geometries obtained by MRI imaging, which estimated elevated cyclic strains at the apex (6%–14%) and the external-common carotid adjoining wall (5%–11%)—both common sites of early inflammation—as compared to 3%–5% strain in regions distal to the bifurcation.^24^

Experiments using cultured ECs have demonstrated that cyclic stretch plays a role in every stage of atherogenesis.^25–27^ Chien describes stress fiber alignment perpendicular to the direction of uniaxial cyclic stretch as anti-atherogenic [Fig. 2(a)] based on the correlation between the time course of alignment and c-jun N-terminal kinase (JNK) activation, with JNK activation subsiding as stress fiber aligns.^28^ Switching the direction of cyclic uniaxial strain after the cells have aligned resulted in a new round of JNK activation as the cells realigned in response to the new stretching direction. Equibiaxial cyclic strain induced sustained JNK activation, however, since the stress fibers are unable to reorient in a direction that avoids strain [Fig. 2(b)]. Analogous to multidirectional WSS experiments, cyclic uniaxial strain applied with either alternating directions of stretching or randomly chosen directions resulted in increased pro-inflammatory NF-κB signaling relative to that measured in ECs subjected to unidirectional cyclic strain.^29^

### Additional insight into soft substrates

Elevated pulse pressure is typically associated with arterial stiffening that occurs with age, metabolic disorders, and atherosclerosis.^30^ Culturing ECs on hydrogels with stiffnesses matching that of young and aging intima promotes an atherogenic EC phenotype as exemplified by decreased barrier integrity and enhanced leukocyte transmigration.^31^ Increased matrix stiffness enhances EC spreading and stress fiber formation by providing additional resistance to cell pulling forces.^32^ There is limited information regarding the distribution of stiffness within the arterial tree. Probing the stiffness of ECs in intact rabbit aorta by atomic force microscopy revealed that stiffness is approximately twice as high at the medial wall of the iliac bifurcation as compared to the abdominal aorta immediately upstream of the bifurcation.^33^

The majority of studies exploring the role of WSS and cyclic stretch has involved culturing cells that are relatively rigid glass (E ∼ GPa) or silicone rubber sheets (E ∼ MPa). Recent studies revealed that the response of ECs to applied forces depends on substrate stiffness. ECs subjected to WSS on compliant hydrogels (E ∼ kPa) elongate to a greater extent, have tighter cell junctions, and have lower RhoA activation and greater eNOS and extracellular signal-regulated kinase (ERK) phosphorylation than when subjected to WSS on more rigid substrates.^34^ ECs subjected to cyclic uniaxial strain on soft collagen gels align parallel to the direction of stretch, while the same cells align perpendicular to stretch on more rigid collagen coated silicone rubber membranes.^35^

Measurements of traction forces using compliant substrates provide important insight into the contractile state of ECs. A transient stretch induces a rapid drop in smooth muscle cell stiffness and traction force that recovers over hundreds of seconds^36^ though this remains to be shown in other cell types such as ECs. Slow cyclic strain of ECs results in an initial drop in traction and recovery of traction after 100 min once the cells aligned their shape and traction field perpendicular to the direction of stretching.^37^ Cell–cell stresses and intracellular stresses were measured in confluent EC monolayers using a novel 3D inter-/intracellular force microscopy technique based on 3D traction force microscopy.^38^ Steady WSS generated a preferential increase in intracellular and junction tensions along the direction of flow, while oscillatory WSS did not. Steward *et al.*^39^ demonstrated that steady WSS caused a decrease in traction force and the direction of traction forces aligned with the direction of flow within 1 h, which preceded cell alignment observed after 12 h. Thus, while traction forces align as would be expected based on stress fiber reorientation in response to cyclic strain and WSS, these changes in traction forces occur before significant remodeling is observed. The authors speculated that cytoskeletal fluidization and resolidification occurs early in response to application of WSS to result in the reorientation of traction forces though the details of this process remain to be demonstrated.

## NEGATIVE FEEDBACK LOOPS IN EC REMODELING

The common theme in the response to ECs to different types of WSS and cyclic strain is the importance of cell alignment relative to the direction of stress/strain. Further, both WSS and cyclic strain are responsible for causing EC alignment. This suggests a need for a framework by which to understand how the mechanical forces synergistically results in the EC orientations found at different locations in the arterial tree and how these orientations contribute to the ability of the ECs to adapt to their dynamic mechanical environment.

**FIG. 3.**
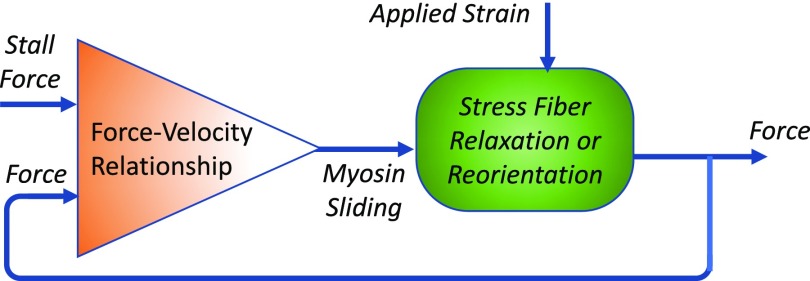
Negative feedback loop for cyclic stretch-induced cytoskeletal remodeling. Cyclic stretching of individual stress fibers results in a deviation of a fiber tension from the stall force of individual myosin II filaments. The myosin II filament compares the fiber tension with the stall force to respond by concentric (when the tension is below the stall force) or eccentric sliding (when the tension is above the stall force) to minimize the deviation in tension.

One way to consider the effects of WSS and cyclic strain on EC alignment, or lack of, is in the context of a negative feedback loop (Fig. 1) as has been used to understand homeostatic regulation of physiological processes. To illustrate, consider the following model for cyclic strain-induced EC alignment based on controlling tension in stress fibers (Fig. 3). The assembly of stress fibers and development of a basal tension (*u*t) occur through actomyosin crossbridge cycling (see the review in Ref. 40 for details). Externally applied strain provides a disturbance (*u*d) to stress fiber tension and compensatory sliding of myosin along actin filaments (*u*c) in the direction, which acts to re-establish the tension at the stall force for myosin *u*t. The number of actomyosin crossbridges is inversely proportional to the velocity of sliding, and hence, rapid sliding increases the probability of stress fiber disassembly.^41^ Reassembly of the stress fiber in a direction that reduces the applied strain on the stress fiber would thus act as another mechanism for the Effector to adjust stress fiber tension. This model has two main predictions for ECs subjected to cyclic uniaxial strain: (1) strain rates that result in rapid changes in tension sensed at the actomyosin crossbridges exceeding the rate of compensatory dissipation of tension perturbations via actomyosin crossbridge cycling result in stress fiber disassembly in the direction of strain and accumulation of stress fibers oriented in the perpendicular direction; (2) low strain rates that result in slow changes in tension result in dissipation of tension disturbances via crossbridge cycling and hence no stimulus for stress fiber disassembly and realignment. These results are consistent with experimental results.^32–44^

It should be noted that other models of stretch-induced stress fiber alignment have been proposed, which also predict perpendicular alignment of the fibers. These models can be broadly classified as being based on actin polymerization^45–47^ or focal adhesions dynamics.^48,49^ Indeed, it is likely that stretch-induced remodeling depends on all these molecular events. A number of other molecules have been identified as playing roles in mechanotransduction, which are classified as sensors, controllers, and effectors. Mechanosensors include integrins, focal adhesion proteins, caveolae, primary cilia, growth factor receptors, G-proteins, and glycocalyx. There is debate as to which mechanical measures (e.g., stress, strain, and strain rate) serve as the parameter *u* being regulated, and there are likely multiple mechanisms of regulation.

**FIG. 4.**
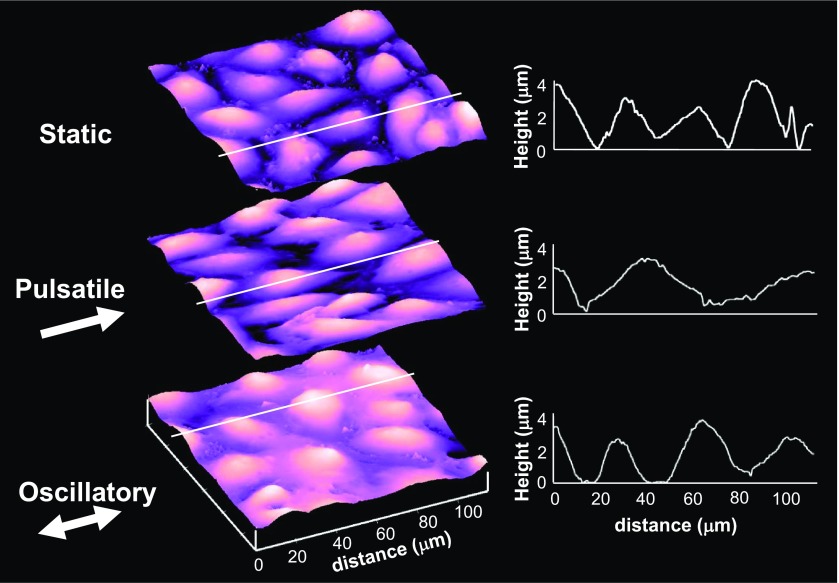
Effects of directional and non-directional WSS on EC shape. Pulsatile WSS (1.2 ± 0.6 Pa) for 12 h induces EC elongation in the direction of flow, effectively streamlining the cell shape. Oscillatory WSS (0 ± 0.6 Pa) does not change the cell shape relative to ECs in static culture.

As illustrated in Fig. 4, pulsatile (or steady) WSS induces EC elongation in the direction of flow, while oscillatory WSS does not change the EC morphology relative to ECs in static culture. Despite the attention WSS has received, there are relatively few models reported to describe WSS-induced EC remodeling. Civelekoglu-Scholey^50^ proposed a model that assumes a gradient in Rac1 activation peaking at the downstream edge and transient global RhoA deactivation that disassembles stress fibers after the onset of WSS. The polarized Rac1 activation causes cell elongation in the downstream direction, followed by recovery of RhoA activity and stress fiber reassembly in the direction of cell elongation. It is speculated that the Rac1 activation gradient is due to mechanical asymmetry and directional integrin treadmilling preferentially at the downstream edge and cell elongation along the direction of flow, which is consistent with experimental observations.^51^ Allen *et al.*^52^ used computational modeling to explicitly describe the mechanical asymmetry caused by steady WSS to result in downstream Src and Rac activation. They further predicted that cell elongation parallel to the flow direction reduces the gradient in mechanical asymmetry, while elongation perpendicular to the direction of flow increases this gradient. These model predictions suggest that ECs that are not aligned in the direction of flow are stimulated to elongate in the direction of flow, but the resulting cell elongation dampens this signal and thus results in a new mechanical steady-state condition. Ferko *et al.*^53^ used computational modeling to demonstrate focusing of stresses at focal adhesions in cells subjected to WSS, thus magnifying the effects of WSS at putative sensors. Their model also predicted a local gradient in stresses, with upstream tensile and downstream compressive stresses computed in the vicinity of individual focal adhesions (FAs). While it remains to be shown, it can be reasoned that periodic changes in the direction of flow will cause greater fluctuations in the stresses acting on FAs than would occur with steady or pulsatile flow. Together, these studies suggest that mechanical asymmetry is sensed by an EC (Comparator) and this triggers EC elongation (Effector) and that this remodeling serves to minimize asymmetrical force distribution and thus cause the output to approach the setpoint (*u → u_t_*).

As noted above, intracellular signaling plays an important role in WSS- and stretch-induced EC alignment. The role of a complex of endothelial-specific proteins localized at cell–cell junctions, consisting of PECAM-1, VE-cadherin, and VEGF receptor in WSS-induced alignment is well studied.^54–56^ WSS increases tension on PECAM-1 and triggers association with the vimentin cytoskeleton and activation of Src and downstream activation of VEGFR, PI3K, and eNOS. PI3K also activates integrins, which mediates cell alignment in directional WSS and activation of inflammatory pathways in disturbed flow.^54^ The extent, and even the direction, of cyclic strain-induced stress fiber alignment is highly dependent on the Rho GTPase and Myosin Light Chain Kinase pathways that regulate actomyosin activity.^44,57^ Blocking stretch-activated calcium channels, such as TPV4, inhibits stretch-induced EC reorientation.^58,59^ In addition to integrins, focal adhesion proteins such as Src are also implicated in the stretch reorientation response.^60^

In the context of negative feedback regulation, these models suggest that remodeling of the cell to reduce asymmetry in stresses at the upstream and downstream edges of the cell would serve to reduce the disturbance that induces further remodeling. Another less obvious prediction of these models is that a disturbance lacking asymmetry would not result in alignment, and hence would not allow the cell to reduce the effects of the disturbance. Such non-asymmetric disturbances include multidirectional or oscillatory WSS and equibiaxial cyclic strain, which do not induce EC alignment despite the continuous application of a mechanical stimulus.

## CONCLUSION

The past 15 years have not only supported the feedback mechanisms described in Chien's Cannon Lecture but also provided important insights that expand upon these concepts. The common theme remains that mechanical forces that disturb mechanical homeostasis contribute to a pro-atherogenic EC phenotype. The recognition that multidirectional WSS plays a significant role has motivated the identification of new metrics to correlate with the localization of atherosclerotic lesions in the arterial tree. Given the importance of cyclic strain, the spatial distributions of strain magnitude, orientation, and anisotropy need to be mapped. While the expected direction of EC alignment caused by the local WSS and cyclic strain is well matched in straight regions, mismatches that are more likely to be identified at bifurcations and curvatures may correlate with regions prone to lesions.

Computational models have been used to predict changes in stress fiber tension that are currently challenging to measure directly. Such models can aid in interpretation of data, such as correlating changes in stress fiber tension with the time course of stretch-induced signal transduction.^40^ Thus, negative feedback models predicting the remodeling of ECs in response to their local mechanical environment provide a tool for linking the ability (or lack thereof) to adapt to these forces to mechanotranduction events relevant to vascular disease. Cell contractility plays a key role in the EC response to WSS, cyclic strain, matrix stiffness, and mechanotransduction, indicating the potential insights from models that consider both internally generated and externally applied forces in the overall behavior of ECs, potentially leading to new therapies for cardiovascular disease.^61^
